# Practical Notes and Formulæ

**Published:** 1888-04

**Authors:** 


### PRACTICAL NOTES AND FORMULÆ.

**Glycerole for Pigmentary Spots on the Skin.**—Dr. Unna prescribes the following :

℞. Oxide of bismuth........................<br>

Rice starch.............................aa. ij grs.<br>

Kaolin.................................. iv grs.<br>

Simple glycerole.......................... x grs.<br>

Distilled rose-water, q. s.

Put t.is mixture, by means of the forceps, on the pigmentary spots and let it dry. Bathe carefully before making the application.—*Le Clinique.*

**Diarrhea and Dysentery.**—(*Medical Brief.*)

℞. Bismuth Sub. Nit...............................ʒ iv,<br>

Tinct. Capsici..................................ʒ j,<br>

Kennedy's Ext. Pinus Can. (dark)..............ʒ iv,<br>

Papine .........................................ʒ jss,<br>

Syr. Zingiberia.................................ʒ ij,<br>

Aquæ, add to make...............................℥ iij.

M. Sig.: Teaspoonful every hour till relieved. Shake well before using.

**Delirium Tremens.**—(*Medical Brief.*)

℞. Tinct. Capsici.................................℥ ss,<br>

Bromida.........................................℥ j,<br>

Celerina........................................℥ ijss.

M. Sig.: Teaspoonful, in water, as required, for wakefulness and excitement.

**Indigestion.**—

℞. Pepsini (Jensen)...............................ʒ iij,<br>

Acidi Tartarici.................................grs. v,<br>

Glycerini.......................................℥ jss.<br>

Vini Xerici ad..................................℥ viii.

M. Sig.: Teaspoonful after each meal.

**Cough Mixture.**—A good one :

℞. Spiritus Etheris Nitrosi.......................ʒ iv,<br>

Vini Ipecac.....................................ʒ vi,<br>

Papine..........................................ʒ ij,<br>

Syr. Tolu.......................................℥ ijss,

M. Sig.: Teaspoonful three or four times a day.

3

**Cholera Morbus of Malaral Fever.**—Prof. A. B. Palmer, Ann Arbor:

℞. Camphor gum..............................
Opium ..................................aagr. j,
Calomel.... ............................gr. iij,
Sugar of milk....................... .......gr. xv.
M.

Rub up into a very fine, impalpable powder. This should be dropped into a teaspoonful of water, and taken far back into the mouth, followed by a single, small swallow of water. Repeat in an hour if relief does not follow.—*Ib.*

**Syphilis in the Early Stages.**—Prof. Ricord, Paris:

℞. Hydrarg. protoid..............................gr. $\frac{1}{8}$–$\frac{1}{2}$,

Ext. gentianæ..................................q. s.

Ft. Pil. No. 1. Sig.—One thrice daily after meals.—*Ib.*

**A New Treatment for Tape Worm.**—I have had within the last three years several cases of tape worm to treat, and finding such strong objection to the large draught of medicines in ordinary use, I prescribed the following:

℞. Chloroform................................ .....

Ext. male fern..............................aaʒ j.

Emul. ol. ricinni (50 per cent.)................ʒ iiij.

Sig.—All to be taken at once after twenty four hours' fast.

In every case the medicine was well borne and the worm expelled entire. In two cases I omitted the male fern and the result was the same as when the latter drug was in combination. My object in reporting this treatment is to induce others of the profession to try the chloroform and report results. I claim for this agent a specific and rational action as an adjuvant in the expulsion of the worm. It anæsthetizes or suspends vitality, and any active purge during anæsthesia of the tænia is all that is necessary to expel it. I earnestly ask those who have cases to try the chloroform, or chloroform and male fern, as above prescribed, and report results.—*Medical and Surgical Reporter.*

**Fetid Bronchitis.** — A case of extremely fetid bronchitis, which before treatment was expectorating one and one-half pints in twenty-four hours, was in a month's time cured, so that only half an ounce was expectorated in the same length of time; the agent used was oil of sandal wood, and was prescribed by Prof. Da Costa, in 5 drop doses, to be taken three times daily; afterward increased to five times.—*Ib.*

**Prof. Da Costa**, in a case of *polyuria*, gave fluid extracts of ergot in half drachm doses three times daily. The cause was traced to grief occurring some time previously.—*Ib.*

